# Nasolacrimal Canal Topography in Relation to the Maxillary Position: CBCT Insights into Le Fort Osteotomy and Fixation Safety

**DOI:** 10.3390/diagnostics15233008

**Published:** 2025-11-26

**Authors:** Mehmet Emre Yurttutan, Merve Berika Kadıoğlu, Mahzun Yıldız, Ömer Faruk Kocamaz, Meyra Durmaz, Mehmet Alp Eriş, Anıl Kamal

**Affiliations:** 1 Department of Oral and Maxillofacial Surgery, Faculty of Dentistry, Ankara University, 06560 Ankara, Türkiye; mzunyildiz22@gmail.com (M.Y.); omerf.kocamaz@gmail.com (Ö.F.K.); mehmet.alp.eris@gmail.com (M.A.E.); anl.kamal37@gmail.com (A.K.); 2 Department of Orthodontics, Faculty of Dentistry, Ankara University, 06560 Ankara, Türkiye; mkadioglu@ankara.edu.tr (M.B.K.); dtmeyradurmaz@gmail.com (M.D.)

**Keywords:** nasolacrimal canal, Le Fort osteotomy, nasolacrimal duct, orthognathic surgery

## Abstract

**Background/Objectives**: Le Fort I osteotomy is a widely performed surgical procedure for correcting maxillary deformities, but it carries the risk of rare complications, including nasolacrimal duct (NLD) injury. This study evaluated the anatomical relationship between the nasolacrimal canal (NLC) and the maxilla across different skeletal patterns via cone-beam computed tomography (CBCT) to define safer zones for fixation during orthognathic surgery. **Methods**: This retrospective study included 76 patients (152 canals) scheduled for orthognathic surgery. The participants were classified into retrognathic, orthognathic, and prognathic groups based on SNA values. Four linear distances were measured on sagittal CBCT sections: from the superior (SL), middle (ML), and inferior (IL) points of the NLD to the anterior maxillary border and from the canine apex to the inferior NLC point (IC). A total of 608 measurements were analyzed via ANOVA, the Kruskal–Wallis test, and post hoc tests, with significance set at *p* < 0.05. **Results**: The ML distance was significantly greater in the prognathic group than in the retrognathic and orthognathic groups (*p* < 0.001). The IL distance was significantly shorter in retrognathic individuals (*p* < 0.001). No significant differences were found in SL (*p* = 0.063) or IC (*p* = 0.141) among the groups. **Conclusions**: The proximity of the NLC to the maxilla varies according to the skeletal pattern. The retrognathic maxilla results in shorter IL distances, suggesting increased risk during fixation, whereas the prognathic maxilla results in greater ML distances. Preoperative CBCT-based individualized evaluation is recommended to optimize fixation and reduce NLD injury risk in Le Fort I osteotomy.

## 1. Introduction

Orthognathic surgery is an interdisciplinary treatment modality undertaken jointly by oral and maxillofacial surgeons and orthodontists in patients presenting with dentofacial deformities who have reached, or are nearing, skeletal maturity [1,2]. Orthognathic surgery is a well-established and effective treatment option for the correction of maxillofacial deformities. Among the available procedures, the Le Fort I osteotomy remains the most commonly utilized and reliable technique for the management of skeletal maxillary deformities [3,4]. The technique that facilitates mobilization of the maxilla is generally associated with predictable and favorable outcomes. Nevertheless, Le Fort I osteotomy has a distinct risk profile. These complications have been extensively documented in the literature and include unfavorable fractures, hemorrhagic complications, infection, bone necrosis, nasal septum deviation, neurosensory disturbances, malunion, and nonunion [5,6]. The incidence of complications related to Le Fort I osteotomies has been reported to range between 4% and 9% [7].

Nasolacrimal duct (NLD) obstruction, which may lead to postoperative nasal discomfort and permanent impairment of nasolacrimal function, is a very rare complication that has been reported in only a limited number of cases in the literature [8,9]. Intraoperatively, the distal ostium of the NLD and the anterior wall of the lacrimal sac are especially vulnerable to injury. The risk of NLD involvement is increased in procedures such as superior repositioning of the maxilla, inferior turbinectomy, osteosynthetic screw placement, and high-level Le Fort I osteotomy techniques [3].

After Le Fort I osteotomy, the use of long miniplates or fixation screws in the piriform aperture region may increase the risk of iatrogenic perforation when the screws are placed near the NLD. This risk is particularly pronounced with superiorly positioned screws of excessive length, which may penetrate the duct and consequently disrupt the tear drainage system, resulting in postoperative hemolacria. Accordingly, careful selection of the screw length in accordance with anatomical constraints, along with consideration of the NLD trajectory during fixation, is essential for minimizing the likelihood of such complications [10,11]. In some cases, even in the absence of direct trauma, nasolacrimal duct obstruction may result from secondary inflammatory changes induced by indirect injury rather than from direct damage to the duct itself [12,13].

The aim of this retrospective study was to evaluate the anatomical position of the nasolacrimal canal (NLC) and the surrounding bone thickness in patients scheduled for Le Fort I osteotomy, thereby contributing to the prevention of potential intraoperative complications. For this purpose, measurements were obtained from cone-beam computed tomography (CBCT) images to assess the distances from different regions of the NLC to the anterior margin of the maxilla and to the canine root across various maxillary morphology groups (retrognathic, orthognathic, and prognathic). The findings are expected to facilitate safer planning of fixation screw and plate placement during Le Fort I osteotomies, reduce the risk of NLD injury, and promote a more controlled and complication-free surgical procedure.

## 2. Materials and Methods

### 2.1. Study Design and Ethics Approval

This study was designed as a retrospective investigation and was conducted by evaluating CBCT images archived at the Department of Oral and Maxillofacial Surgery, Faculty of Dentistry, Ankara University. Ethical approval was obtained from the Ethics Committee of the Faculty of Dentistry, Ankara University (Decision Date: 8 September 2025, Decision No: 13/6). The study was carried out in accordance with the ethical principles of the Declaration of Helsinki (2013). All participants were informed about the study both verbally and in writing, and voluntary informed consent forms were obtained.

### 2.2. The Study Sample

Individuals aged 18–50 years who had completed skeletal development, presented with maxillary canines in situ, and were scheduled for orthognathic surgery without any congenital anomalies (such as syndromes or cleft lip and palate) were included in the study. Individuals with radiographs containing artifacts or with pathologies such as cysts or tumors were excluded.

**Figure 1:**
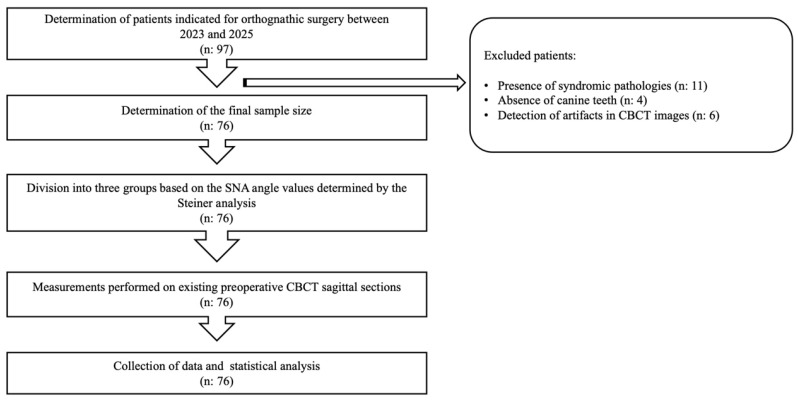
Flow diagram illustrating the study design and sample selection process. CBCT: Cone-beam computed tomography, SNA: Sella-Nasion-A point angle.

This study was designed to include a total of 97 patients who were diagnosed with orthognathic surgery indications at the Department of Oral and Maxillofacial Surgery, Faculty of Dentistry, Ankara University, between 2023 and 2025. However, according to the inclusion criteria, 11 patients with syndromic pathologies, 4 patients with missing canine teeth, and 6 patients with artifacts detected in their CBCT images were excluded. Consequently, the final sample size was 76 patients (Figure 1). The sample size of the study exceeded the minimum number determined by power analysis (*n* = 66; power = 0.80, α = 0.05) and was therefore considered to have sufficient statistical power.

A total of 152 canals from 76 individuals were divided into three groups on the basis of the SNA angle values determined via Steiner analysis. Those with an SNA value less than 80° were assigned to the retrognathic group, those between 80° and 85° to the orthognathic group, and those 85° or greater to the prognathic group.

- Group 1 (retrognathic maxilla): 30 individuals—60 lacrimal canals
- Group 2 (orthognathic maxilla): 22 individuals—44 lacrimal canals
- Group 3 (prognathic maxilla): 24 individuals—48 lacrimal canals

### 2.3. Measurements

All measurements were performed on the sagittal slice that provided the clearest visualization of both the inferior opening of the nasolacrimal canal at the inferior meatus and the superior opening at the orbital floor. The slice in which these two anatomical points were simultaneously visible with optimal clarity was selected for standardized evaluation. The junction of the nasolacrimal canal with the inferior meatus was designated as the inferior point (I), while the site where the canal opens into the orbit was identified as the superior point (S). The middle point (M) was determined by calculating the midpoint between these two anatomical landmarks.

**Figure 2:**
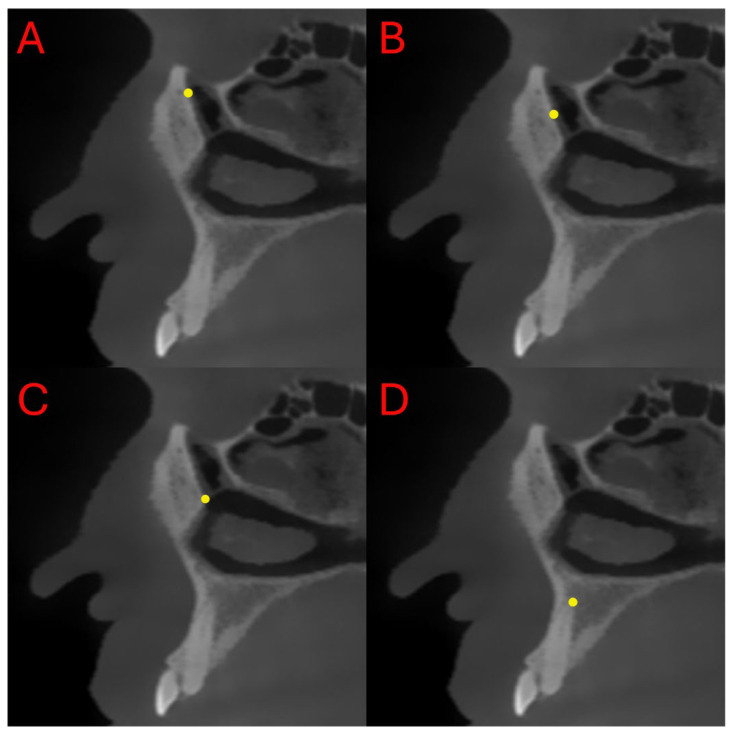
Representative sagittal CBCT images showing the measurement points (**A**–**D**) defined at different anatomical levels of the NLD. (**A**) S—Superior ostium of the NLD; (**B**) M—Middle point of the NLD; (**C**) I—Inferior ostium of the NLD; (**D**) C—Level of the canine apex.

**Figure 3:**
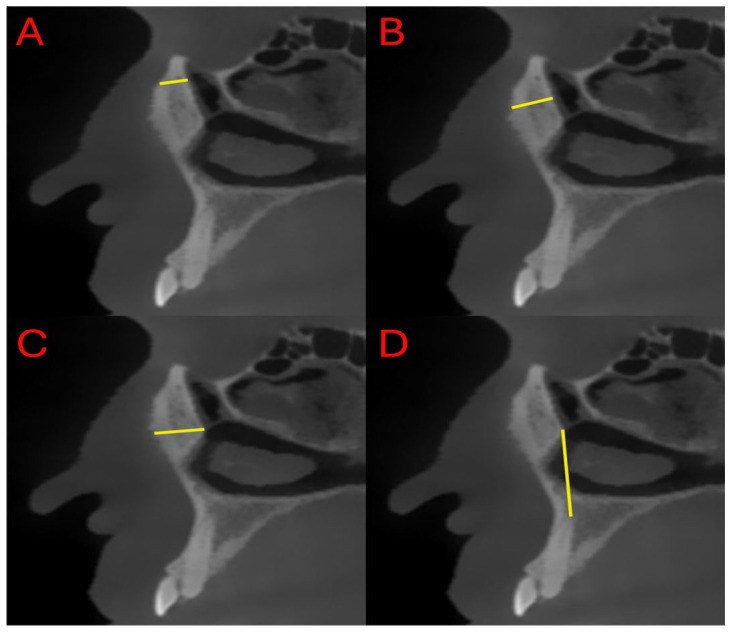
Representative sagittal CBCT images showing measurements of the distance between the NLD and the maxilla. (**A**) Distance between the superior ostium of the NLD and the anterior maxillary border; (**B**) distance between the middle point of the NLD and the anterior maxillary border; (**C**) distance between the inferior ostium of the NLD and the anterior maxillary border; (**D**) distance between the canine apex and the inferior ostium of the NLD.

During the measurements, the following four parameters were evaluated (Figure 2 and Figure 3):

- Distance between the superior ostium (S) of the nasolacrimal canal and the anterior maxillary border (SL)
- Distance between the middle point (M) of the nasolacrimal canal and the anterior maxillary border (ML)
- Distance between the inferior ostium (I) of the nasolacrimal canal and the anterior maxillary border (IL)
- Distance between the canine apex (C) and the inferior ostium (I) of the nasolacrimal canal (IC)

The four selected measurements were designed to evaluate the relationship of the NLC with the maxilla at different levels to demonstrate the potential contact of plates and screws with the duct during Le Fort I osteotomy and thereby anatomically define surgical risk zones. A total of 608 measurements were performed in this study.

### 2.4. Measurement Repeatability and Reliability

All the measurements were performed by a single experienced researcher. To assess measurement reliability, the same anatomical reference points on each participant’s CBCT images were measured twice with a two-week interval. Agreement between the two sets of measurements was evaluated via the intraclass correlation coefficient (ICC). The ICC analysis was conducted via a two-way, absolute-agreement model, and values greater than 0.90 were obtained for all four measurement parameters. These results indicate that the measurements were highly repeatable and reliable.

### 2.5. Imaging Protocol

Imaging procedures were performed via a NewTom 7G CBCT unit (NewTom, Verona, Italy) with a 29 cm × 30 cm field of view (FOV). Dose adjustments were made by a single technician according to the patient’s body type. All the scans were carried out in accordance with the ALARA (As Low As Reasonably Achievable) principle. Automatic exposure parameters were used with a slice thickness of 1 mm. CBCT scanning was performed with the patient in an upright position, with the head stabilized via a head support device. During CBCT acquisition, patients were positioned in the supine position. The Frankfort horizontal plane was adjusted to be perpendicular to the floor, and the midsagittal plane was aligned with the patient’s facial midline using the glabella as the primary reference point. This positioning ensured a standardized head orientation for all scans prior to image reconstruction and measurement. Standard radiation protection protocols routinely implemented in the clinic were applied. Following reconstruction of the images with a voxel size of 0.600 mm, axial, sagittal, and cross-sectional slices were measured via Synapse software v7.2.000 (Fujifilm Corporation, Tokyo, Japan).

### 2.6. Statistical Analysis

The data obtained in this study were analyzed via the IBM SPSS Statistics version 22 (IBM Corp., Armonk, NY, USA). The normality of the data was assessed with the Kolmogorov–Smirnov test. The relationships between categorical variables were examined via the chi-square test. For comparisons among groups, the Kruskal–Wallis H test was applied to nonnormally distributed variables, whereas one-way ANOVA was used for normally distributed variables. For multiple comparisons, Dunn–Bonferroni and Tukey HSD tests were performed. A significance level of 0.05 was adopted; results with *p* < 0.05 were considered statistically significant, whereas those with *p* > 0.05 were considered not significant.

## 3. Results

The relationship between sex and the skeletal pattern was statistically significant (*p* = 0.005). Retrognathic and orthognathic patterns were more frequently observed in females, whereas the prognathic pattern was more common in males. This finding suggests that sex may be an influential variable in skeletal morphology. Specifically, the tendency toward maxillary prognathism in males and the predominance of retrognathic features in females may be explained by sex-related anatomical differences (Table 1).

The mean age of the participants was 32 ± 6.57 years, and no statistically significant difference in age distribution was observed among the groups (*p* > 0.05) (Table 2).

No statistically significant difference was observed among the groups regarding the distance between the superior point of the NLC and the anterior maxillary border (*p* = 0.063). The analysis revealed that the SL values presented similar distributions across all the groups examined (Table 3).

A statistically significant difference was observed in the ML values (*p* < 0.001). Compared with both the retrognathic and orthognathic groups, the prognathic group presented significantly greater ML values. Although no statistically significant difference was found between the orthognathic and retrognathic groups, the values were relatively greater in the orthognathic group (Table 4).

A statistically significant difference was observed among the groups in terms of the IL values (*p* < 0.001). Compared with the other groups, the retrognathic group presented significantly lower IL values. Although no statistically significant difference was found between the orthognathic and prognathic groups, the values were relatively lower in the orthognathic group (Table 4).

No statistically significant difference in the IC values was detected among the groups (*p* = 0.141) (Table 4).

## 4. Discussion

This retrospective study aimed to compare the anterior bony distances of the NLC among three skeletal patterns (retrognathic, orthognathic, and prognathic), classified according to SNA values, via CBCT scans of patients scheduled for orthognathic surgery. The study sample was determined with adequate statistical power following the application of the inclusion and exclusion criteria, and analyses were conducted on scans acquired under a standardized imaging protocol.

The SNA angle is one of the most widely used cephalometric indicators for assessing the anteroposterior position of the maxilla relative to the cranial base [14,15]. In the literature, sexual dimorphism in the anteroposterior position of the maxilla, as measured by SNA, has generally been reported to be statistically insignificant, whereas maxillary linear dimensions (particularly Co-A and ANS-PNS) are consistently greater in males. A comprehensive review further confirmed that most studies did not identify significant sex-related differences in SNA but demonstrated that Co-A tends to be greater in males [16]. Nevertheless, some population-based studies have reported greater SNA values in females than in males [17]. This heterogeneous pattern indicates that the SNA does not demonstrate a consistent sex-related trend, although maxillary dimensions are generally greater in males. In the present study, the retrognathic maxilla was more frequently identified in females than in males, whereas the prognathic maxilla was more prevalent among males.

Although NLD injury is rare following Le Fort I osteotomy, it may lead to clinical outcomes such as epiphora, transient or permanent nasolacrimal obstruction, and even hemolacria. The underlying mechanism is generally described as superior placement of plates or screws around the piriform aperture, compression adjacent to the duct, or indirect damage associated with postoperative edema [10,18,19]. Even without primary injury to the NLD, secondary impairment may arise from plates or screws placed in close proximity through mechanisms such as compressive effects, hardware migration, or inflammation and subsequent fibrosis. These processes may ultimately lead to epiphora, NLD obstruction, and/or dacryocystitis [20]. The morphological structure of the nasolacrimal duct and the valve of Hasner can show considerable anatomical variation among individuals, and these structures may also be influenced by factors such as age and sex [21,22,23]. Therefore, the extent to which the nasolacrimal duct is affected after Le Fort I osteotomy, as well as the resulting clinical outcomes, may vary from patient to patient. Shoshani et al. reported the development of epiphora in a single patient during the second week after surgery [24]. In a series of 54 patients who underwent high Le Fort I osteotomy reported by Keller and Sather, epiphora was observed in only one patient, and it was transient [25]. Jang et al. reported epiphora in a total of 10 patients between January 2011 and July 2012 [18]. Freihofer and Brouns reported that NLD injury was observed in approximately 4% of patients who underwent surgery [26]. Another study documented the development of hemolacria in a patient, caused by perforation of the NLD by a 7 mm screw placed during fixation [11]. In this context, the anterior maxillary cortical distance to the NLD should be considered alongside screw depth and angulation when evaluating the risk of duct injury. In our series, these distances varied significantly according to the skeletal pattern, with the ML distance increasing in prognathic cases and the IL distance decreasing in retrognathic cases, whereas no significant differences were observed in the SL or IC distances.

In a study on a Le Fort I population that directly measured the distance between the NLC and the external maxillary cortex in the sagittal plane, Kaba et al. reported a mean distance of 7.18 ± 3.15 mm at the inferior level of the duct [7]. In the present study, the IL distance in the retrognathic group was significantly lower than that in both the orthognathic and the prognathic groups. At the same level, the mean values were 6.91 mm in the prognathic group, 6.58 mm in the orthognathic group, and 5.92 mm in the retrognathic group. The ML distances were measured as 6.26 mm, 5.83 mm, and 5.42 mm, respectively. The shorter distances observed in the retrognathic group may indicate a relatively greater likelihood of complications during fixation screw–plate placement. Therefore, the use of 5 mm screws for fixation near the middle and inferior margins of the duct may help reduce the risk of potential injury. Although the IL and ML distances are statistically significantly lower in retrognathic patients, these differences are not thought to have a significant clinical effect in most cases. However, the relatively closer proximity of the nasolacrimal duct to the maxilla in these skeletal patterns suggests an increased likelihood of complications due to secondary damage, particularly in fixations performed at these levels. Therefore, this potential risk should be considered when planning fixation in retrognathic cases. Owing to its proximity to the orbit, the superior margin of the duct is generally avoided for fixation. However, when this approach is unavoidable, the risk of duct injury may increase considerably.

In clinical practice, the osteotomy line in Le Fort I procedures is typically planned with reference to the canine root apex at a level positioned at least 4–5 mm above the root apices [27,28]. In a cadaveric simulation study, You et al. reported that the high Le Fort I osteotomy line was, on average, ~5.2 mm from the inferior orifice of the NLD. They further noted that the inferior point of the duct was situated approximately 4.4 mm superior and 14.6 mm posterior to the inferior turbinate [29]. In high-level Le Fort I osteotomies, the risk of nasolacrimal structure injury is elevated, whereas in standard-level cuts, canine guidance provides a reliable reference. Several studies in the literature have also reported cases involving substantial maxillary impaction [30,31]. In such scenarios, greater amounts of maxillary impaction increase the proximity to the NLD, thereby increasing the risk of duct injury, particularly during fixation. In the present study, the mean distance between the canine apex and the inferior border of the NLC (IC) was 20.07 ± 2.51 mm, with no statistically significant intergroup differences (*p* = 0.141). This finding suggests that the canine–canal relationship may serve as a relatively consistent reference point, independent of the skeletal pattern. In surgical planning, both the targeted degree of impaction and the fixation stage should consider the maxillary–canal distance, and the potential risk of duct injury should be evaluated on a patient-specific basis. For cases involving proximity to the inferior or middle canal margins, the use of 5 mm screws may minimize the likelihood of injury. Accordingly, preoperative CBCT-based individualized measurements are recommended to delineate safe zones and optimize screw placement strategies.

### Strengths and Limitations

The present study has several notable strengths. It was retrospectively conducted on a homogeneous sample, and all measurements were obtained from high-resolution CBCT scans acquired under a standardized protocol. Repetition of the measurements twice by a single examiner yielded excellent reproducibility (ICC > 0.90), further enhancing the reliability of the data. Moreover, the sample size was determined to be sufficient on the basis of power analysis, supporting the robustness of the statistical findings. While only a few prior studies have evaluated the distance between the NLC and the external maxillary surface, to our knowledge, no comprehensive investigation has previously assessed these measurements across different skeletal patterns and reference points. Thus, this study provides a valuable contribution to the literature. Nonetheless, several limitations must be acknowledged. Its single-center, retrospective design may limit the generalizability of the results. In addition, the study focused solely on preoperative anatomy, and postoperative changes in the nasolacrimal canal following maxillary movement could not be assessed. The CBCT examinations were obtained with a voxel size of 0.6 mm, which is acceptable for clinical use but may not fully capture subtle variations in canal dimensions; confirming these measurements with higher-resolution CBCT protocols would help determine whether the observed differences are anatomical or related to imaging resolution. In this study, only the bony structure of the nasolacrimal canal was evaluated. Future research using contrast-enhanced CT will be necessary to include the nasolacrimal duct itself and to investigate postoperative changes in both the canal and its soft-tissue component. In addition, prospective multicenter studies with larger cohorts would help confirm and expand the present findings.

## 5. Conclusions

This study demonstrated that the distances between the NLC and the maxilla vary according to the skeletal pattern. The IL values were significantly shorter in retrognathic individuals, whereas the ML values were significantly greater in prognathic individuals. In contrast, no significant differences were observed in the SL and IC values. While the use of 5 mm screws for fixation near the middle and inferior levels of the canal may help reduce the risk of complications, the likelihood of duct injury should be carefully considered when fixation is performed closer to the superior level. Moreover, the IC values were found to be independent of the skeletal pattern and may serve as a stable reference point; taking this measurement into account in cases requiring substantial maxillary impaction could contribute to reducing the degree of complication risk. These findings also highlight that preoperative CBCT-based individualized measurements may enhance patient safety and improve the predictability of surgical planning in Le Fort I osteotomy.

## Figures and Tables

**Table 1:** Sex distribution by skeletal pattern group.

		Group								Chi-Square Test	
		Retrognathic		Orthognathic		Prognathic		Total		χ^2^	*p*
		*n*	%	*n*	%	*n*	%	*n*	%		
Sex	Female	38	63.3	30	68.2	18	37.5	86	56.6	10.6	0.005
	Male	22	36.7	14	31.8	30	62.5	66	43.4		
	Total	60	100.0	44	100.0	48	100.0	152	100.0		

**Table 2:** Age distribution by skeletal pattern group.

		Group						Kruskal–Wallis H Test			
		*n*	Mean	Median	Minimum	Maximum	SD	Mean Rank	H	*p*	Post Hoc
Age	Retrognathic	60	27.77	26	15	55	8.30	83.23	6.7	0.054	-
	Orthognathic	44	24.00	25	18	34	3.92	62.05			
	Prognathic	48	26.63	26	18	37	5.52	81.33			

SD: standard deviation.

**Table 3:** Statistical evaluation of SL distance (mm).

		Group						Kruskal–Wallis H Test			
		*n*	Mean	Median	Minimum	Maximum	SD	Mean Rank	H	*p*	Post Hoc
SL	Retrognathic	60	3.91	3.83	2.36	5.79	0.75	84.95	5.5	0.063	-
	Orthognathic	44	3.63	3.51	2.75	5.33	0.56	64.40			
	Prognathic	48	3.76	3.69	2.75	4.84	0.52	77.03			

SD: standard deviation, SL: distance between the superior ostium of the NLC and the anterior maxillary border.

**Table 4:** Statistical evaluation of the ML, IL, and IC distances (mm).

		Group					One-Way ANOVA		
		*n*	Mean	Minimum	Maximum	SD	F	*p*	Post Hoc
ML	Retrognathic	60	5.42	3.23	8.61	1.34	9.09	0.0001	3-1 3-2
	Orthognathic	44	5.83	3.84	7.34	0.81			
	Prognathic	48	6.26	4.87	8.17	0.62			
IL	Retrognathic	60	5.92	3.37	8.73	1.12	14.3	0.0001	1-2 1-3
	Orthognathic	44	6.58	4.66	8.18	0.94			
	Prognathic	48	6.91	5.17	8.69	0.80			
IC	Retrognathic	60	19.68	13.86	24.27	2.31	1.98	0.141	-
	Orthognathic	44	19.99	15.48	26.02	2.74			
	Prognathic	48	20.64	15.16	25.91	2.50			

SD: standard deviation, ML: distance between the middle point of the NLC and the anterior maxillary border, IL: distance between the inferior ostium of the NLC and the anterior maxillary border, IC: distance between the canine apex and the inferior ostium of the NLC.

## Data Availability

The data presented in this study are available upon request from the corresponding author. However, the data are not publicly available due to patient privacy and ethical restrictions.

## Abbreviations

The following abbreviations are used in this manuscript:

ALARA	As low as possible achievable
ANOVA	Analysis of variance
CBCT	Cone-Beam Computed Tomography
FOV	Field of view
ICC	Intraclass correlation coefficient
IC	Distance between the canine apex and the inferior ostium of the NLD
IL	Distance between the inferior ostium of the NLD and the anterior maxillary border
ML	Distance between the middle point of the NLD and the anterior maxillary border
NLD	Nasolacrimal duct
SD	Standard deviation
SL	Distance between the superior ostium of the NLD and the anterior maxillary border
SNA	Sella-Nasion-A point angle
